# Draft genome sequence of *Bifidobacterium thermophilum* CCRC isolated from chicken faeces

**DOI:** 10.1128/mra.00845-25

**Published:** 2025-10-29

**Authors:** Sinalo Mani, Tshifhiwa Paris Mamphogoro

**Affiliations:** 1 Gastrointestinal Microbiology and Biotechnology Unit, Animal Production Institute, Agricultural Research Council 72748, Irene, Pretoria, South Africa; The University of Arizona, Tucson, Arizona, USA

**Keywords:** genome assembly, probiotic, *Bifidobacterium thermophilum*, poultry microbiota, gut health

## Abstract

This study reports the draft genome sequence of *Bifidobacterium thermophilum* strain CCRC, isolated from chicken faeces. This strain has potential probiotic properties. The draft genome is 2,260,057 bp and fragmented into 23 contigs.

## ANNOUNCEMENT

*Bifidobacterium thermophilum* is a thermophilic, Gram-positive bacterium commonly found in the gastrointestinal tracts of various animals, including poultry (1, 2). It is considered a core member of the gut microbiota and is frequently isolated from the distal gut, contributing to intestinal homeostasis (3). Its presence has been associated with health-promoting effects, such as enhancing immune responses and improving intestinal barrier function by competing with pathogenic bacteria (4, 5). For instance, supplementation of *B. thermophilum* in poultry diets has been linked to improved growth performance and gut health, highlighting its probiotic potential (4, 6).

The faecal samples were collected from chickens housed at the ARC Poultry Unit in Irene, South Africa (GPS: −25.904, 28.216), maintained for conservation purposes. The bacterial strain was cultured in de Man, Rogosa, and Sharpe (MRS) media (Oxoid, UK), as previously described by Khurajog et al. (7) and preserved in the GI Microbiology in-house Biobank on agar slants below −130°C. The culture was revived in de Man, Rogosa, and Sharpe (MRS) broth (Oxoid, UK) supplemented with 0.05 g/L cysteine-HCl (cysHCl) and incubated anaerobically using an Anaerobic Gas Pack (Oxoid) for 24–48 h at 37°C. Single colonies were obtained by subculturing onto MRS agar plates and then inoculated to MRS broth for DNA extraction.

Genomic DNA from pure cultures was extracted using a Bacterial Miniprep Kit (Zymo Research, Irvine, CA, USA). DNA concentration and quality were evaluated using a NanoPhotometer NP80 (Implen GmbH, Munich, Germany) and a Qubit Fluorometer (Thermo Fisher Scientific, MA, USA). Whole genome sequencing was conducted on the DNBSEQ-G400 MGI platform (MGI Tech, Shenzhen, China). Paired-end sequencing (2 × 150 bp) generated 4,449,734 high-quality reads, achieving ~590× coverage. Quality control was assessed using FastQC v0.72 (http://www.bioinformatics.babraham.ac.uk/projects/fastqc), and low-quality reads were trimmed using Trimmomatic v0.36 (8), retaining bases with phred scores of 30 or higher for 90% of the read lengths. The reads were assembled *de novo* using SPAdes v3.15.3 (9), and assembly quality was evaluated using QUAST v5.0.2. Genome completeness was assessed using CheckM v1.0.18 (10) and estimated at 100% family level (10).

The resulting draft genome comprised 2,260,057 bp across 23 contigs (N50 = 146,093 bp), with a GC content of 60.05%. Genome annotation was carried out using both Prokka and PGAP. Prokka v1.14.5 (11) was used to annotate the located version of the genome and identified 1,758 total genes, of which 1,712 were protein-coding. The publicly available NCBI assembly was annotated with PGAP (12). Gene counts reported here are consistent with the PGAP annotation. Taxonomic assignment using GTDB-Tk v2.3.2 (13) confirmed the isolate as *Bifidobacterium thermophilum*. The genome shared 99.99% average nucleotide identity (ANI) with *Bifidobacterium thermophilum* strain RBL67 (GCF_000347695.1) GCA_000771265.1, as calculated by FastANI v0.1.3 (14), confirming its affiliation to the *Bifidobacterium thermophilum* taxon. Secondary metabolite biosynthesis gene clusters were evaluated using antiSMASH v6.0.0 (15). PGAP was used to identify genes related to probiotic functions, including those involved in bile salt tolerance, adhesion, acid tolerance, stress tolerance, and immunomodulation (Table 1). Default parameters were used for all software unless otherwise specified.

**TABLE 1:** Summary of probiotic genes within the genome of *Bifidobacterium thermophilum* CCRC using PGAP predictions

Gene	Function	NCBI protein accession no.	Annotation
GNAT	Bile salt tolerance	MGG6303044.1	GNAT family N-acetyltransferase
*bsh*		MGG6304356.1	Choloylglycine hydrolase
*tuf*	Adhesion	MGG6303098.1	Elongation factor Tu
LPXTG		MGG6303711.1	LPXTG cell wall anchor domain-containing protein
*pgi*		MGG6303850.1	Glucose-6-phosphate isomerase
*gap*		MGG6304180.1	Type I glyceraldehyde-3-phosphate dehydrogenase
*gro*L	Acid tolerance	MGG6303135.1	Chaperonin GroEL
*Gro*ES		MGG6303774.1	Co-chaperone GroES
*atp*B		MGG6304496.1	F0F1 ATP synthase subunit A
*atp*E		MGG6304497.1	ATP synthase F0 subunit C
*atp*A		MGG6304500.1	F0F1 ATP synthase subunit alpha
*atp*D		MGG6304502.1	F0F1 ATP synthase subunit beta
		MGG6304503.1	F0F1 ATP synthase subunit epsilon
*Nrd*H	Stress tolerance	MGG6303166.1	Glutaredoxin-like protein NrdH
*trx*B		MGG6303333.1	Thioredoxin-disulfide reductase
*tpi*A		MGG6303399.1	Triose-phosphate isomerase
*dna*J		MGG6303406.1	Molecular chaperone DnaJ
*Clp*X		MGG6303644.1	ATP-dependent Clp protease ATP-binding subunit ClpX
*trx*A		MGG6304275.1	Thioredoxin
*Lys*M	Immunomodulation	MGG6304251.1	LysM peptidoglycan-binding domain-containing protein

## Data Availability

This whole-genome sequencing project has been deposited at NCBI under the BioProject number PRJNA1262373. The version described in this paper is the first version, with a genome accession number of GCA_050565525.1. The SRA accession number is SRR33598564, and the BioSample accession number is SAMN48452950.
